# The impact of COVID-19 and associated lockdowns on traumatic spinal cord injury incidence: a population based study

**DOI:** 10.1038/s41393-023-00939-6

**Published:** 2023-11-02

**Authors:** Euan J. McCaughey, Frederick K. Ho, Daniel F. Mackay, Jill P. Pell, Peter Humburg, Mariel Purcell

**Affiliations:** 1 Queen Elizabeth National Spinal Injuries Unit, Queen Elizabeth University Hospital, Glasgow, G51 4TF UK; 2 Scottish Centre for Innovation in Spinal Cord Injury, Glasgow, UK; 3 Neuroscience Research Australia, Randwick, NSW 2031 Australia; 4 School of Medical Sciences, University of New South Wales, Kensington, NSW 2052 Australia; 5 College of Medicine, Veterinary & Life Sciences, University of Glasgow, Glasgow, G12 8QQ UK

**Keywords:** Epidemiology, Neurophysiology

## Abstract

**Study design:**

Natural experiment

**Objectives:**

To determine whether COVID-19 restrictions were associated with changes in the incidence of traumatic spinal cord injury (TSCI) in Scotland.

**Setting:**

The Queen Elizabeth National Spinal Injuries Unit (QENSIU), the sole provider of treatment for TSCI in Scotland.

**Methods:**

Time series analysis of all admissions for TSCI between 1st January 2015 and 31st August 2022.

**Results:**

Over the 8-year study period, 745 patients were admitted to the QENSIU with a TSCI. Interrupted time series analysis showed that level 3 and 4 COVID-19 lockdown restrictions (the most severe levels) were associated with lower incidence of TSCI (RR 0.63, CI% CI 0.47, 0.82, *p* < 0.001). The associations were stronger in people aged over 45 (additive interaction *p* = 0.001), males (additive interaction *p* = 0.01) and non-tetraplegia (additive interaction *p* = 0.002). The incidence of TSCI due to deliberate self-harm was higher (0.41 versus 0.23 per month) during restrictions.

**Conclusions:**

Overall, TSCI incidence reduced in Scotland when lockdowns were implemented, presumably due to lower engagement in risky activities. The increase in TSCI due to deliberate self-harm may reflect increased mental health problems and social isolation and should be anticipated and targeted in future pandemics. The change in incidence during the COVID-19 pandemic may have an economic impact and see a temporary reduction in the burden on health and social care. The results of this study will be useful for resource planning in future pandemics.

## Introduction

A spinal cord injury (SCI) is a devastating event, with a global incidence rate of over 200,000 cases per year [1]. These injuries have a massive impact on both the person with the SCI and their families, with an estimated lifetime cost of care of £1.12 million in the United Kingdom [2]. Increasing our understanding of the epidemiology of SCI enables the development and targeting of prevention strategies, accurate evaluation of novel treatments and planning of immediate and long-term care provision [3].

There is a rapidly growing interest in the epidemiology of SCI, and the temporal trends associated with this, with five papers published in ‘Spinal Cord’ on this topic alone between January 2020 and August 2021 [4–8]. The COVID-19 pandemic, and associated lockdowns, impacted hospital admission patterns for a wide range of common conditions [9]. A change in the incidence of SCI caused by COVID-19 is likely to have broad and long-term implications; including economic impact and burden on health and social care.

Since 1994, all new traumatic spinal cord injuries (TSCIs) in Scotland have been treated solely at the Queen Elizabeth National Spinal Injuries Unit (QENSIU), Glasgow. A clinical database has been in operation since this time, collecting clinical and demographic data about every new admission. As this encompasses every new TSCI in Scotland, data from the QENSIU can be used to reliably assess changing trends in TSCI epidemiology at a population level [10]. Notably, despite the stresses placed on the healthcare system by the COVID-19 pandemic, there was no impact on the ability of the QENSIU to admit all patients with a TSCI during this period. We hypothesise that COVID-19 lockdowns, and the associated limitations on road traffic and outdoor/sporting activities, would be associated with reduced levels of TSCI. The aim of this study was to evaluate changes in the incidence of TSCI in Scotland during the COVID-19 pandemic to inform healthcare planning and improve the management of future pandemics.

## Methods

### Study setting

The QENSIU is Scotland’s sole centre for treating TSCI. Information about the Unit’s creation and funding has been previously described [10]. In 2019, the last full calendar year prior to COVID-19 reaching the UK, 41% of patients were admitted to the QENSIU within 48 h of injury, with 66% of all patients admitted within one week [11]. It is assumed all TSCIs that occur in Scotland will eventually be admitted to the QENSIU. Details of the aetiology, gender, age injury level and severity of all new admissions are entered into the QENSIU database. The database does not include information about people who die due to TSCI prior to admission.

Approval for the collection and evaluation of data within the database was granted by the Data Custodian for the Queen Elizabeth University Hospital, Glasgow, Scotland.

### Participants

All patients admitted to the QENSIU during the study period (1st January 2015 to 31st August 2022) were ascertained from the clinical database. The neurological level of injury and degree of impairment after TSCI was assessed by a Consultant in Spinal Injuries on admission and defined according to the International Neurological Classification of Spinal Injury using the American Spinal Injury Association Impairment Scale (AIS) [12]. Patients who were neurologically intact, and who were recorded as an AIS E on admission, were excluded along with those under 16 years of age. TSCI aetiology was classified in accordance with the International SCI core data set as assault, fall, transport, sports and leisure (including cycling for consistency with previous work [10] and falls that occurred during sporting activities, such as rock and mountain climbing) and other traumatic (deliberate self-harm (DSH), iatrogenic [13], and industrial) [14].

### Analyses

**Fig. 1:**
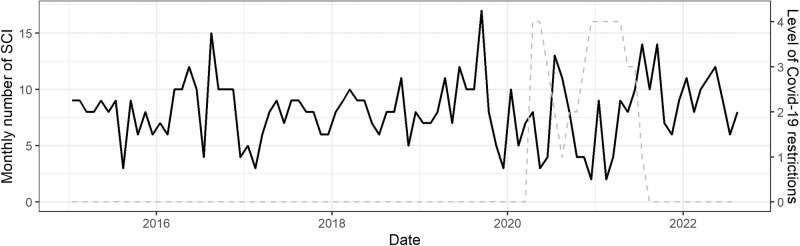
Monthly incidence of traumatic spinal cord injury in Scotland between January 2015 and August 2022. Periods of COVID-19 associated lockdowns are shown in grey dashed line. Level 0 = no lockdown measures. Level 1 = Restrictions on indoor meetings between households (maximum of 6 people from 2 households). Level 2 = As level 1, plus no indoor meeting with other households with restrictions also placed on outdoor meetings (maximum of 6 people from 2 households). Level 3 = As level 2, plus no alcohol sales indoors and outdoors with hospitality venues all to close by 6 pm. Level 4 = As level 3, plus closure of all non-essential shops, hospitality venues and gyms. The population is encouraged to only leave home for essential reasons (shopping, health care appointments etc). Equivalent to a full population-level lockdown.

All data were analysed according to the level of restrictions placed on the public at that point in time. Scotland saw four different levels of lockdown applied during the COVID-19 pandemic, with Level 1 being the least severe and Level 4 being the most severe (equivalent to a full population-level lockdown) [15]. These levels are summarised in Fig. 1. January 2015 to 31st March 2020 inclusive were denoted as the pre COVID-19 period, while September 2021 to August 2022 was classified as the post COVID-19 period. Incidence per million was calculated by comparing the incidence of TSCI in a calendar year with the corresponding years midyear population estimate [16]. As data was not yet available for 2022, the 2021 midyear population estimate was also used for 2022.

Poisson regression models were used to examine the number of monthly TSCIs, adjusted for age, sex, year, season, tetraplegia, and injury completeness. The level of Covid-19 was the primary exposure variable. There is no evidence of over-dispersion. The model residual deviance/degrees of freedom was 1226.4/1462 = 0.84. This indicates mild under-inflation which could lead to slightly conservative standard error estimates. Year and month were initially modelled as penalised cubic splines in the generalised additive model framework as a preliminary analysis. The model revealed linear association for a year and non-linear association for a month (Supplementary Figure. 1). The Month variable was categorised into seasons (December-February; March-May; June-August; September-November) based on the inflection points in the spline. The exposure variable was collapsed into a binary variable: No restriction to level 2; level 3 and 4 restrictions. Additive interactions between Covid-19 restrictions and: age (<45 vs. >=45), sex (female vs. male), tetraplegia, and complete injury were examined using relative excess risk due to interaction (RERI). Under this model, we estimated the counterfactual number of SCI cases as if level 3 and 4 restrictions did not occur. These, compared with the observed SCI cases, were used to estimate the number of reduced SCI cases due to COVID-19. Incidence was also analysed with a supplementary exposure variable of COVID-19 Stringency Index [17]. This score is a measure of lockdown level summated over 9 domains - school closures; workplace closures; cancellation of public events; restrictions on public gatherings; closures of public transport; stay-at-home requirements; public information campaigns; restrictions on internal movements; and international travel controls. The index is calculated as the mean score of the nine metrics, each taking a value of between 0 and 100. A higher score indicates a stricter response (i.e. 100 = strictest response). If policies vary at the subnational level, the index is shown as the response level of the strictest sub-region. The incidence of deliberate self-harm was described in different periods since there was not sufficient power to conduct a formal analysis. R Statistical Software (version 4.2.2) was used with packages mgcv and interactionR. Descriptive data are presented as means plus standard deviations or 95% confidence intervals [14, 18]. Incidence rate ratio (IRR) and 95% CI were used to infer associations and their corresponding precision.

## Results

### Overall impact of COVID-19

There were 510 patients admitted to the QENSIU with a TSCI over the 73 months Pre-COVID-19, compared to 121 during the 16-month COVID-19 period and 114 in the 12 months post COVID-19. This corresponds to an annual incidence of 18.0 per million population over the entire study period [16]. The characteristics of people admitted to the QENSIU with TSCI during the study is shown in Table 1.

**Table 1:** Incidence rates and demographics of traumatic spinal cord injury in Scotland between 1^st^ January 2015 and 31^st^ August 2022.

Annual Incidence	97.9 ± 12.0
Annual incidence rate (per million)	18.0 ± 2.2
Age (years)	55.6 ± 19.0
Males (%)	72.8 ± 3.5
Cervical (%)	69.1 ± 3.7
AIS A (%)	25.8 ± 3.0
Aetiology (%)	
Fall	65.5 ± 3.5
Transport	14.8 ± 2.1
Sports and Leisure	10.0 ± 2.8
Assault	1.4 ± 1.6
Other	8.3 ± 3.0

Results are presented as the mean, with the standard deviation shown in brackets. *AIS* ASIA Impairment Scale.

### Effects of lockdown measures

A time series plot on the monthly number of TSCI is shown in Fig. 1. Preliminary analysis found no association between level 1 and 2 restrictions and no restriction periods (Supplementary Table 1). Level 3 and 4 restrictions were associated with a lower incidence of TSCI (RR 0.63, CI% CI 0.47, 0.82, *p* < 0.001) (Table 2). The effect of level 3 and 4 restrictions was stronger in people aged over 45 (additive interaction *p* = 0.001) and slightly stronger in tetraplegia (additive interaction *p* = 0.002). There is a strong sex difference in the effect of level 3 and 4 restrictions to TSCI (additive interaction *p* = 0.01), where the reduction in TSCI incidence is also observed in males.

**Table 2:** Association between Level 3 and 4 Covid-19 lockdown restrictions and incidence of traumatic spinal cord injury compared to incidence during no and level 1 and 2 restrictions. RR: Relative Risk RERI: relative excess risk due to interaction.

	RR/RERI	95% CI^1^	*p*-value
Overall (RR)	0.63	0.47, 0.82	<0.001
Age			
<45	0.85	0.55, 1.33	0.11
>=45	0.55	0.76, 0.39	<0.001
RERI	−1.09	−1.78, −0.39	0.001
Sex			
Female	1.96	0.82, 4.74	0.13
Male	0.99	0.65, 1.49	0.95
RERI	−1.00	−0.15, −1.85	0.01
Tetraplegia			
No	0.62	0.45, 0.84	0.002
Yes	0.67	0.38, 1.20	0.18
RERI	0.30	0.09, 0.51	0.002
Complete injury			
No	0.65	0.48, 0.88	0.006
Yes	0.58	0.34, 0.98	0.04
RERI	0.20	−0.03, 0.43	0.07

When comparing with the COVID-19 Stringency Index [17], an alternative method of quantifying lockdown, there was no apparent association between this Index and incidence (IRR per 1 unit of Stringency Index: 0.997, 95% CI: 0.995–1.00, *p* = 0.066).

Level 3 and 4 restrictions were associated with 37 (95% CI 18-62) fewer SCI cases (Table 3). Among these, most were aged >=45 years; male, paraplegic and had incomplete injuries.

**Table 3:** Estimated reduction in SCI cases due to the Covid-19 pandemic. Estimated as the difference between the actual observed SCI cases and the counterfactual as if Covid-19 did not occur. A negative number indicated a reduction in SCI due to Covid-19.

	Number	95% CI^1^
Overall	−37.1	−17.5,−61.7
Age		
<45	−4.8	1.1, −12.2
>=45	−32.4	−18.6, −49.5
Sex		
Female	−1.7	4.3, −9.4
Male	−35.4	−21.8, −52.3
Tetraplegia		
No	−30.7	−15.4, −49.6
Yes	−6.4	−2.1, −12.0
Complete injury		
No	−26.3	−12.1, −43.8
Yes	−10.9	−5.3, −17.9

### Deliberate self harm

During the 5 years Pre-COVID period there were 14 cases of TSCI caused by deliberate self-harm (DSH) in Scotland. There were no TSCIs caused by DSH observed in the first year of COVID. However, in the 3 month period from April-August 2021, representing months 13 to 17 of COVID associated lockdowns, there were seven TSCIs caused by DSH. This corresponds to an incidence rate of 0.41 (95% CI 0.23–0.73) per month during the COVID period, compared to 0.23 (95% CI 0.14–0.36) per month pre COVID. Defying the norms of other causes of TSCI, five of these seven cases were in females (71%), the mean age was 31.2 (10.0) years, only one (14%) resulted in a cervical injury and only two (28%) in a complete injury (1 AIS A, 1B, 2C, 3D).

## Discussion

The findings of this study indicated that the implementation of lockdown measures during the COVID-19 pandemic was associated with a significant reduction in the overall incidence of TSCI. The highest levels of lockdown led to specific reductions in TSCI incidence in males and people older than 45 years of age, with these severe lockdowns also leading to reduced rates of tetraplegia. Of concern, the lockdowns were associated with an increase in TSCI caused by deliberate self-harm, particularly among young women. We postulate that lockdowns restricted activities commonly associated with TSCI, such as road traffic accidents and sporting activities, leading to an overall reduction, but increased mental health problems and social isolation leading to an increase in self-harm. The findings suggested that the benefits and risks to overall TSCI and TSCI due to self-harm respectively were associated with the imposition of any degree of lockdown being imposed. We found no evidence that the stringency of the restrictions (as proxied using the Stringency Index) was related to TSCI incidence. This could possibly be due to the null association between mild restrictions (Levels 1–2).

Our previous research demonstrated that in the twenty-year period from 1994–2014 there was a significant change in the demographics of new TSCIs in Scotland [10]. Specifically, the mean age at injury increased by almost 10 years, there was a significant increase in the number of TSCIs being caused by falls, and there was also a significant increase in incomplete injuries and injuries resulting in tetraplegia. Most of these changes can be explained by considering an ageing population, with associated increased risks of low impact falls, improved driving and workplace safety and a reduction in violent crime. However, the results of this study show that global pandemics are also likely to have a major, if all be it temporary, impact on the demographics of TSCI and that the response to such a pandemic–restrictions, changes to laws etc., can make a direct contribution to this change.

Previous studies have shown that COVID-19 was associated with worse mental health in students, healthcare workers and the general population [19–21]. The COVID-19 pandemic also saw increased rates of suicide attempts [22]. The increase in the incidence of deliberate self-harm seen here is likely to be related to this change in mental health and associated suicide attempts and identifies an area which may need further support in future pandemics. For example, mental health support service could proactively identify and intervene higher risk patients in the primary care system.

### Strengths

This was an unselected cohort of TSCI across the whole of the Scottish population. Access to more than five years of data prior to COVID-19 restrictions provided information on the underlying numbers and time trends. We were able to adjust for a range of potential confounders. The interrupted time series models that we used provided a robust analysis of the population-level data, robust against time-invariant confounders. We were also able to examine the moderation of TSCI characteristics on the associations between Covid-19 restrictions and TSCI risk. We excluded non-traumatic SCI which might be impacted differently by COVID-19 restrictions as lockdown measures are unlikely to have impacted the natural time course of these nontraumatic conditions.

### Limitations

People with a TSCI who died before being admitted to the QENSIU were not included in this study. Therefore, the incidence rates reported here may be an underestimate due to failure to capture peri-accident mortality. However, it is unlikely that the percentage of people dying from TSCI prehospital admission differed in both periods. As with all natural experiments, it is possible that other unknown or unmeasured interventions may have occurred at the same time as the intervention of interest; namely, COVID-19 lockdowns. Finally, due to the relatively low incidence of TSCI it was not practical to stratify by age, sex, injury level or completeness of injury. Larger international collaborations and/or meta-analyses should be conducted to allow sub-group analyses, which would add significant value to the literature.

## Conclusion

Lockdowns associated with the COVID-19 pandemic saw a significant reduction in the overall incidence of TSCI in Scotland. The highest levels of lockdown saw a reduction in TSCI in males and those over 45 years of age, and a reduced incidence of tetraplegia. Concerningly, there was an increase in the incidence of TSCI caused by deliberate self-harm. These changes will have long-term implications for the delivery of health and social care and should inform the management of future pandemics.

### Supplementary information

Supplementary figure legend

Sup Table 1

Sup Fig 1

## Data Availability

Deidentified data used to generate the results of this study are available from the corresponding author upon reasonable request.
