# Supplementary material for: The impact of COVID-19 and associated lockdowns on traumatic spinal cord injury incidence: a population based study
Source: Spinal Cord. 2023 Nov 2;62(1):1–5. doi: 10.1038/s41393-023-00939-6 (PMC10783541; doi:10.1038/s41393-023-00939-6)
Supplement: Supplementary file 1 — Supplementary figure legend [file 41393_2023_939_MOESM1_ESM.docx]

Supplementary Figure 1. Non-linear association of year and month with traumatic spinal cord injury incidence. RR: relative risk
