# Supplementary material for: The impact of COVID-19 and associated lockdowns on traumatic spinal cord injury incidence: a population based study
Source: Spinal Cord. 2023 Nov 2;62(1):1–5. doi: 10.1038/s41393-023-00939-6 (PMC10783541; doi:10.1038/s41393-023-00939-6)
Supplement: Supplementary file 2 — Sup Table 1 [file 41393_2023_939_MOESM2_ESM.docx]

Supplementary Table 1. Preliminary analysis model for traumatic spinal cord injury incidence

Modelled using Poisson regression model

| **Characteristic** | **RR** | **95% CI** | **p-value** |
| --- | --- | --- | --- |
| Level of restrictions |  |  |  |
| 0 | — | — |  |
| 1 | 1.28 | 0.84, 1.96 | 0.3 |
| 2 | 0.90 | 0.60, 1.37 | 0.6 |
| 3 | 0.69 | 0.46, 1.04 | 0.075 |
| 4 | 0.61 | 0.43, 0.87 | 0.006 |
| Age | 2.61 | 2.22, 3.06 | <0.001 |
| Sex | 2.49 | 2.12, 2.92 | <0.001 |
| Tetraplegia | 0.24 | 0.20, 0.29 | <0.001 |
| Complete injury | 0.35 | 0.30, 0.41 | <0.001 |
| Year (penalised spline) |  |  | 0.2 |
| Month (penalised spline) |  |  | 0.015 |
