# Supplementary figures and images for: The impact of COVID-19 and associated lockdowns on traumatic spinal cord injury incidence: a population based study
Source: Spinal Cord. 2023 Nov 2;62(1):1–5. doi: 10.1038/s41393-023-00939-6 (PMC10783541; doi:10.1038/s41393-023-00939-6)

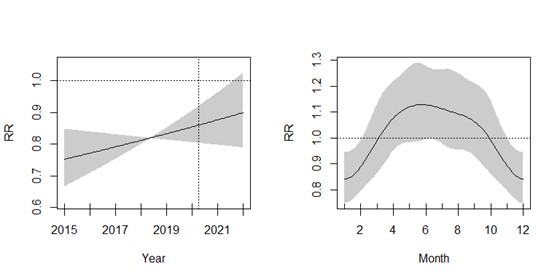

Supplement: Supplementary file 3 — Sup Fig 1 [file 41393_2023_939_MOESM3_ESM.jpg]
